# Elimination of lymphatic filariasis as a public health problem from the Cook Islands

**DOI:** 10.1186/s41182-018-0094-9

**Published:** 2018-05-15

**Authors:** Charlie Ave, D Ramaiah Kapa, Eric Ottesen

**Affiliations:** 1 From the Public Health Department, Community Health Service Directorate, Ministry of Health, Government of Cook Islands, Rarotonga, Cook Islands; 2 Pondicherry, 605008 India; 3 Neglected Tropical Diseases Support Center, Taskforce for Global Health, 325 Swanton Way, Decatur, GA 30030 USA

**Keywords:** Lymphatic filariasis, Elimination, PacELF, Cook Islands

## Abstract

**Background:**

The Cook Islands has a long history of high-endemicity lymphatic filariasis (LF) transmitted by *Aedes* vector mosquitoes. Though the infection prevalence had declined between 1975 and 1999 following episodic treatment activities, still infection was widespread with pockets of persistent infection. Beginning in 1999, the Cook Islands embarked on a national program, in partnership with Pacific Programme to Eliminate LF (PacELF), to eliminate LF as a public health problem.

**Methods:**

All 12 inhabited islands were identified as endemic, and six rounds of mass drug administration (MDA) with once-yearly, single-dose albendazole plus diethylcarbamazine (DEC) were implemented during 2000–2006 to interrupt transmission of LF. Surveys carried out at the baseline, mid-term, stop-MDA, and post-MDA periods assessed LF antigen (Ag) prevalence in children and adults. Historical data, health workers’ observations, and hospital records were used to assess the trend and burden of chronic disease.

**Results:**

The baseline Ag prevalence (1999) ranged from 2.0% in Manihiki to > 18.0% in Aitutaki, Mitiaro, and Pukapuka, and the national average Ag prevalence was 8.6%. MDA, carried out with a national treatment coverage over six annual rounds of MDA ranging from 63.5 to 96.7% in different years, was stopped in 2007. By then, the national Ag prevalence had declined to 0.27%. The post-MDA surveillance survey results (2013–2014) showed that Ag prevalence had fallen to 0% in 11/12 islands, and the national prevalence was only 0.03%. Chronic filarial disease had almost entirely disappeared.

**Conclusion:**

The Cook Islands met all the criteria required for the World Health Organization (WHO) to acknowledge elimination of LF as a public health problem, as it did officially in 2016. This success also confirms that LF, even when transmitted by *Aedes* mosquitoes that are recognized to be more efficient than other vector species, can be eliminated as a public health problem by six rounds of MDA.

## Background

Lymphatic filariasis (LF) has been recognized for decades as a significant public health problem in the countries of the South Pacific, with 16 countries in the region, including the Cook Islands, still endemic in 2000 [1, 2]. The epidemiology of LF in the region is notable for (i) its transmission by the day-biting *Aedes* mosquito species that are more efficient than other mosquito vectors of LF [3] and (ii) its distribution of infection being very widely scattered among the islands and atolls of different countries of the region. These epidemiological characteristics make interruption of transmission and elimination of infection particularly challenging.

While some of the Pacific Island countries attempted control of the disease in the past, the results were mixed. However, the launching of the Global Programme to Eliminate Lymphatic Filariasis (GPELF) by World Health Organization (WHO) in the year 2000 [4] provided a unique platform to coordinate a major regional initiative—the Pacific Programme to Eliminate Lymphatic Filariasis (PacELF)—to eliminate the disease from the region [2]. PacELF is a network of 22 Pacific Island countries, formed in 1999 under the umbrella of Division of Technical Support, Western Pacific Region, WHO, with a common objective of elimination of LF from the region, using the preventive chemotherapy strategy of once-yearly distribution of single-doses of two medicines (albendazole (ALB) plus diethylcarbamazine (DEC)) distributed using a mass drug administration (MDA) strategy recommended by the WHO. The Ministry of Health (MOH) of the Cook Islands, accordingly, launched its national program to eliminate LF in the year 2000. The goals, objectives, activities, and impact of the program are presented in this paper.

### Demographics of the Cook Islands

**Fig. 1:**
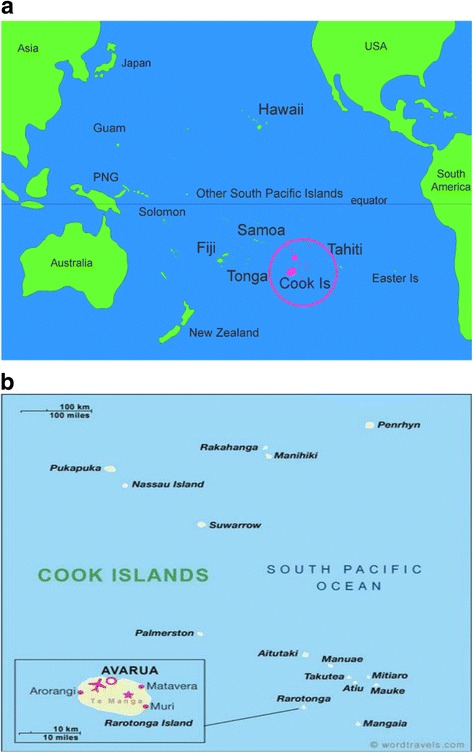
**a** Geographic location of the Cook Islands; **b** individual islands of the Cook Islands

The Cook Islands comprise 15 islands in the middle of the South Pacific Ocean, between Tonga to the west and the Society Islands to the east (Fig. 1a). The islands (Fig. 1b) are divided into a southern group and a northern group. The southern group of islands includes (i) Aitutaki, (ii) Atiu, (iii) Mangaia, (iv) Manuae, (v) Mauke, (vi) Mitiaro, (vii) Palmerston, (viii) Rarotonga, and (ix) Takutea. Rarotonga is the largest island (67 km^2^) and the political and economic epicenter of the country. The remote northern group islands are situated more than 1250 km away from the capital and include (i) Manihiki, (ii) Nassau, (iii) Tongareva (Penrhyn), (iv) Pukapuka, (v) Rakahanga, and (vi) Suwarrow. The islands collectively have a total land area of 240 km^2^.

The census of 2011 indicated a total population of 17,794 and a resident population of 14,974. About 74% of the population lived in Rarotonga, 20% in the other southern group of islands, and 6% in the northern group. The Cook Islands made tremendous progress in the social, economic, and health sectors during the last two decades and is currently classified as an upper middle-income country. The gross national income per capita was $14,918 in 2012 (https://data.un.org/ CountryProfile.aspx?crName= Cook%20Islands), one of the highest in the region. Life expectancy was high at 74 years and infant mortality was low at an average figure of 12 per 1000 live births over the 5-year period (2005–2009), and these parameters are among the best in the Pacific region. Adult literacy is high, and secondary school enrolment rates exceed 90%. Health services in the Cook Islands are provided through a system of child welfare clinics, dental clinics, health centers, a general hospital, and a few private clinics on the main island of Rarotonga. The Community Health Services Directorate is responsible for providing accessible and equitable health care services in the community setting. As of September 2012, Cook Islands had approximately 294 medical staff and nurses.

### Epidemiology of LF

LF in the Cook Islands is caused by aperiodic *Wuchereria bancrofti* (McKenzie1925; Lambert 1926, cited in Sasa [1]) and transmitted by *Aedes polynesiensis*. The breeding habitats of vector species include tin cans, bottles, and coconut shells that are continually filled with water, due to > 100 in. annual rainfall. Transmission of LF mainly occurs in and around houses. Vector infection rates of 9.0 to 25.9% were recorded in the past, but no detailed studies have been carried out after 1960.

LF was widespread across all islands, and its prevalence was higher in the southern group of islands than in the northern group, where microfilaria (Mf) rates up to 40% was recorded in some islands. Steel et al. [5] reported an Ag prevalence of 49.4% in 1975 in Mauke and a hydrocele prevalence of 13.3%. Prevalence of elephantiasis was mostly around 4.0% in different islands studied over the years (Table 1).

**Table 1:** The Mf rate and disease rate reported in various studies in the Cook Islands

Place	Year	Number of people examined for Mf	Mf rate/Ag rate (%)	Number of people examined for chronic disease	Elephantiasis rate (%)^#*^	Author
Southern group of islands						
Rarotonga	1925	197	2.0	197	40.1	Mckenzie 1925^#^
Aitutaki	1925	71	5.6	71	50.7	Mckenzie 1925^#^
Atiu	1926	–		900	2.4	Lambert 1926^#^
Mauke	1926	–	46.2	560	4.1	Lambert 1926^#^
Mitiaro	1926	–	54.8	180	0	Lambert 1926^#^
Rarotonga	1926	–	35.5	–	–	Lambert 1926^#^
Mangaia	1926	–	26.0	–	–	Lambert 1926^#^
Aitutaki	1926	–	49.1	–	–	Lambert 1926^#^
Aitutaki	1949	240	42.5	–	–	Davies 1949^#^
Rarotonga	1957	554	23.3	554	4.3	Iyengar 1957^#^
Aitutaki	1957	1297	20.9	1297	3.7	Iyengar 1957^#^
Rarotonga	1959	1890	22.3	358	2.9	McCarthy 1959^#^
Aitutaki	1959	609	29.2	528	4.2	McCarthy 1959^#^
Aitutaki*	1969*	2492	0.8	–	–	Review of sub-periodic filariasis in South Pacific, 1980
Aitutaki*	1971*	2600	0.2	–	–	Review of sub-periodic filariasis in South Pacific, 1980
Mauke	1975	376	33.2	459	2.6	Weller et al. 1982
Mauke	1975	376	49.4 Ag rate	180	13.3% Hydrocele rate	Steel et al. 2001
Mitiaro Rarotonga	1977	273	18.3	–	–	
Rarotonga	1991	5868 (outpatients)	0.9	–	–	Country data, PacELF, 2000
Rarotonga	1992	2664 (outpatients)	1.0	–	–	Country data, PacELF, 2000
Aitutaki	1992	1370	3.2	–	–	Country data, PacELF, 2000
Mauke**	1992**	627	4.3	–	–	McCarthy et al. (1995)
Mauke**	1992**	627	5.4% Ag rate	–	–	Steel and Ottesen, 2001
Northern group of islands						
Pukapuka	1959	218	29.4	–	–	McCarthy 1959^#^
Pukapuka	1965	498	28.4	498	3.8	Iyengar 1965^#^
Penrhyn	1959	274	5.8	–	–	McCarthy 1959^#^
Rakahanga	1959	226	8.4	–	–	McCarthy 1959^#^
Palmerston	1959	69	8.7	–	–	McCarthy 1959^#^
Manihiki	1959	371	19.7	–	–	McCarthy 1959^#^

*Mass drug administration (MDA) in Aitutaki in 1968

**MDA in Mauke and other out islands in 1986

^#^Cited in Sasa [1]

^#*^Irrespective of the degree of swelling, all cases of lymphedema were classified as elephantiasis, whereas only advanced stages are referred to as elephantiasis in the current literature

#### Prior LF control efforts

Several treatment programs were undertaken over the years, with varying degrees of success. Particularly interesting was an LF control program launched in Atiu in the 1960s, when DEC was begun to be administered both to the entire local population at regular, half-yearly intervals and to arriving visitors until 2000. The results were dramatic and the prevalence of LF on Atiu fell to close to zero level. Similarly interesting was an island-wide mass treatment program that was implemented in 1987 in Mauke and many of the outer islands, where the entire population was given DEC (6 mg/kg) for 7 days. This program resulted in the reduction of Mf prevalence from 30% in 1974 to 5% in 1992 in Mauke [6].

## Methods

### Goals and objectives of programme to eliminate LF

In March 1999, at meetings of the Pacific Regional Ministers convened by the Western Pacific Regional Office (WPRO) of the WHO and by the Secretariat of the Pacific Community (SPC), 22 nations with histories of LF agreed to initiate activities to support the 1997 World Health Assembly Resolution calling for the elimination of LF as a public health problem. WHO and SPC, along with donor agencies and private sector drug donations, then launched a program for eliminating LF in all 22 Pacific Island countries and territories—termed PacELF [2] (http://www.wpro.who.int/southpacific/pacelf/en/; WHO, 2006). With guidance from PacELF, the Cook Islands prepared a draft National Plan of Action (1999–2003), with MDA as the principal intervention measure and its implementation in all inhabited islands. When the findings of the baseline A-Survey were available later in 1999, the National Plan of Action was affirmed, and a schedule of MDA and follow-up surveys were developed.

The goal of the LF elimination program was to eliminate the disease as a public health problem for the Cook Islands. The major objectives were to achieve (a) 100% geographic coverage with MDA by the year 2000 and (b) interruption of LF transmission by 2005.

The MOH followed the policy and strategy of the PacELF whose plan of action envisaged (i) mapping of endemic areas; (ii) implementation of at least five rounds of MDA in all islands, using DEC + ALB combination therapy; (iii) conducting a baseline survey (A-Survey) to assess Ag prevalence, using the immunochromatographic (ICT) card test; (iv) implementation of a “B-Survey” after the 3rd round of MDA to assess the progress of the MDA program, followed by a “C-Survey” after the fifth round to determine if MDA can be stopped; and (v) finally, a “D-Survey” to assess if transmission interruption has been achieved [2].

### Determining LF endemicity

The national filariasis team and the Department of Public Health reviewed all the available information, from MOH records and published and unpublished reports, on prevalence and distribution of LF to define the LF endemicity and the requirement for MDA in each island. In addition, per PacELF recommendation, an “A-Survey” was conducted in 9 of the 12 inhabited islands, during June to December 1999, to establish baseline LF prevalence. Endemicity of the other three islands was assessed using historical data.

### MDA program implementation

Plans were made for effective implementation of the MDA program across all 12 islands, with emphasis on achieving as high a treatment coverage as possible. Each of the 12 islands was considered as an implementation unit (IU) for the MDA.

DEC + ALB combination therapy was used in the MDA program, with ALB procured from the donor pharmaceutical company, GlaxoSmithKline (through WHO/PacELF facilitation) and DEC procured by the MOH through a donation from the Japan International Cooperation Agency. Drugs were procured 3–6 months in advance, stored securely, and supplied from Rarotonga (by ship) 3 weeks prior to the distribution date to the northern group of islands and (by air) 1 week in advance to the southern group.

Health personnel were trained prior to the first round of MDA and subsequently when there was an opportunity to combine it with other health program activities such as maternal and child health and immunization for the different islands, every year. Prior to the first round of MDA in 2000, health workers made house-to-house visits and prepared registers with details of the resident population. Rarotonga, the largest island, was divided into 30 operational areas covered by three teams each distributing drugs in one area per day. Drug distribution across the country took about 2 weeks during each round.

Within the communities in each IU, the teams distributed the drugs from central places such as child welfare clinics and public meeting houses and also via house-to-house and workplace visits. All school children were treated in schools. In Rarotonga, drug distribution booths were also established in important gathering places such as the markets. The drug distribution activity was supported by 1 week of an awareness campaign on TV, radio, and newspapers, and through distribution of pamphlets and fliers, and announcements in churches. As much as possible, directly observed treatment was utilized, i.e., drug distribution teams ensured recipients consumed the drug in their presence. The drugs were given at the WHO recommended dosage—DEC at 6 mg/kg body weight and one tablet of ALB (400 mg). Children below 2 years of age, pregnant women, people with serious illnesses or chronic disease conditions, and people above 80 years of age were excluded from MDA. The health workers involved in the drug distribution were given incentives and allowances to meet the costs of food, transportation, and accommodation.

In addition to the MDA program, a “test and treat” strategy was implemented during 2006–2010 period in Pukapuka and Mitiaro where persistent levels of Ag had been found even after six rounds of MDA.

### Epidemiological monitoring, evaluation, and surveillance

The program followed and implemented the monitoring, evaluation, and surveillance surveys (A, B, C, and D) as per the PacELF guidelines.

#### Indicator and diagnostic tool

Ag prevalence was the indicator used to measure the impact of MDA on LF infection prevalence. It was measured using the ICT card (Alere: Binax Now). The tests were conducted by trained staff members, carefully following the guidelines provided by the test manufacturer. The results were recorded on standardized data forms.

#### A-Survey (baseline survey, 1999)

The “A-Survey” was designed to establish a baseline Ag prevalence and was carried out during June to December 1999. For the survey, a 10% sample was targeted to reflect the age- and island distribution of the total Cook Islands’ population. On each island, every fifth household (geographically) was selected, and two to three persons from each household were tested. The choice of individuals was made both on the basis of the person’s volunteering and on the survey’s need for individuals of a certain age to reflect the age distribution of the island/country. Of the 12 inhabited islands, 9 were surveyed. Those islands not surveyed were Palmerston and Nassau (with 0.3 and 0.5% of the total population) and Penrhyn (3.3%), as they were predetermined to be endemic based on historical data.

#### B-Survey (mid-term/effectiveness survey, 2002)

The survey was conducted between October and December 2002, i.e., 3–5 months after the completion of the third round of MDA. A 10% sample was targeted to reflect the age distribution and island distribution of the total Cook Islands population. While the strategy used for A-Survey was followed in Rarotonga, convenience sampling was the predominant strategy used in other islands. The survey was conducted in 11 islands.

#### C-Survey (prestop-MDA survey, 2005)

The survey was conducted in November 2005, i.e., 1 year after the fifth round of MDA was completed. In this survey, a stratified cluster sampling method was followed. The islands were grouped into three evaluation units (EU). The three EUs are Rarotonga, the northern group islands, and the southern group islands. The villages on Rarotonga or the other islands constituted the “clusters.” In each EU, five to six villages were surveyed, from each of which a predetermined number of individuals (between 100 and 300) was sampled. A minimum sample size of 707 individuals per EU had been determined to be required for statistically significant detection of an ICT prevalence of < 1%, which is the minimum target of the MDA program.

Selection of individuals for testing from each cluster was done through randomization, performed by the Cook Islands Department of Statistics. Lists of names of the selected individuals were provided to the teams carrying out the surveys. If a listed individual could not be located, that person was “replaced” by an individual from a “supplementary” list of names again randomly generated by the Statistics Department.

#### D-Survey (stop-MDA survey, 2007)

After the sixth round of MDA in 2006, the stop-MDA survey was implemented in the year 2007 (similar to transmission assessment survey 1 (TAS 1) in the current M & E guidelines of the WHO). Under the survey, 4415 individuals of all age groups from all the 12 inhabited (and MDA-treated) islands were assessed for Ag, using ICT cards.

#### Test and treat strategy in Pukapuka and Mitiaro (2006–2012)

The entire populations of the two islands were tested during 2006–2010, and those found Ag-positive were treated with ALB + DEC. Ag surveys were repeated in 2012 to assess the impact of test and treat strategy.

#### Post-MDA surveillance surveys (May 2013 to August 2014)

The Post-MDA surveillance survey was conducted during May 2013 to August 2014 covering the entire country, and this is similar to TAS 3 in the current surveillance guidelines [7]. The objectives of this survey were to examine (a) the current Ag prevalence levels in the EUs and (b) if the transmission interruption status observed in various islands in 2007 and in Pukapuka and Mitiaro in 2012 is sustained.

The post-MDA surveillance surveys currently recommended by WHO include assessment of Ag in children of 6–7 years, either in schools or in communities [7]. However, due to the small population sizes of some of the islands, surveys involving 6- to 7-year-old children alone will result in an insufficient sample size for robust results. Hence, the survey on children was conducted only in the two largest islands, Rarotonga and Aitutaki, with a population of 10,572 and 1771, respectively. In the former, 6- to 8-year-old children (grades 1–3) were surveyed, and in the latter, 6- to 14-year-old children (all primary and secondary school children) were surveyed. To provide epidemiological evidence for the elimination of LF in the other ten islands, where the population was in the range of 60 to 562, the entire populations were surveyed. Since the school enrolment rate was > 75%, the survey in Rarotonga and Aitutaki was school-based.

### LF morbidity management and disability prevention

In the Cook Islands, the prevalence of chronic disease had shown a decreasing trend for the three to four decades before PacELF began, probably because of reduced transmission and improved living conditions. As a result, chronic disease is no more prevalent in the younger generation. Since the 1980s, the disease has been observed only in older patients whose numbers have dwindled gradually, due to aging. Communities in the Cook Islands are compact, and there is a good rapport and frequent interaction between health workers and people. The health workers are well aware of the people affected with chronic disease conditions such as filarial lymhoedema/elephantiasis. As of now, according to the observations of the health personnel and hospital records, there is only one lymphoedema patient in the Cook Islands and she lives in Mangaia island. She has been given treatment and training in morbidity management. No hydroceles have been reported either.

### Preparation of dossier

After the program met the WHO LF elimination criteria for sustainable interruption of transmission (i.e., through longitudinal observations on Ag prevalence in children and adults, and from implementing MMDP activities), a dossier was prepared in the WHO prescribed format and submitted to WHO for acknowledgement of the validation of elimination of LF as a public health problem.

## Results

### A-Survey and baseline endemicity (1999)

After reviewing all the available data, the MOH determined that all the islands have a history of LF and require intervention measures to achieve national elimination of LF. The A-Survey of 1884 people showed that eight of the nine islands had Ag-positive individuals, with only one island, Atiu, reporting 0% Ag prevalence. The Ag prevalence ranged from 2.00 to 20.00% across the eight islands, with a national prevalence of 9.66% (Table 2). Two other islands—Palmerston and Penrhyn, which had an Mf prevalence of 8.7 and 5.8%, respectively, in 1959 (Table 1)—were declared endemic on the basis of this historical data. One island—Nassau—was declared endemic as most of its residents were from Pukapuka, which was highly endemic. Thus, the entire country with 12 inhabited islands was considered endemic and eligible for the MDA program.

**Table 2:** Details of A-Survey (baseline survey 1999) and B-Survey (mid-term survey 2002)

Island	A-Survey		B-Survey	
	Number of people surveyed	Number of people positive for Ag (%)	Number of people surveyed	Number of people positive for Ag (%)
Aitutaki	200	37 (18.5)	200	8 (4.00)
Atiu	100	0 (0.0)	150	0
Mangaia	95	6 (6.3)	150	0
Mauke	98	2 (2.0)	150	0
Mitiaro	100	20 (20.0)	110	0
Rarotonga	996	92 (9.2)	773	1 (0.13)
Manihiki	100	2 (2.0)	100	0
Nassau	*	–	50	0
Palmerston	*	–	*	–
Penrhyn	*	–	100	0
Pukapuka	95	18 (18.9)	150	3 (2.00)
Rakahanga	100	5 (5.0)	92	0
Total	1884	182 (9.66)	2025	12 (0.59)

*No survey done

### MDA coverage (2000–2006)

In the Cook Islands, six rounds of MDA were implemented over a period of 7 years, i.e., 2000–2006. An assessment of Ag prevalence in 2005 after the completion of the fifth round of MDA showed Ag prevalence above the target threshold level of 1.0%. Hence, one more round of MDA (i.e., sixth round) was implemented in 2006, as per WHO technical advice. The treatment coverage of the eligible population (i.e., individuals ≥ 2 years of age) ranged from 49.3 to 100%. It remained above 80% in all islands in most of the years (Table 3). There were no reports from health centers on any group of people or any village consistently refusing treatment (systematic non-compliance).

**Table 3:** Treatment coverage of eligible population (i.e., > 2 years old) in different MDA implementation units of the Cook Islands

Island (implementation unit)	Evaluation unit	2000	2001	2002	2003	2004	2005	2006	2007
Aitutaki	Aitutaki	76.3	86.5	95.8	94.9	*	No MDA	93.5	MDA stopped
Atiu	Atiu	83.7	87.0	95.8	95.5	*	No MDA	94.2	MDA stopped
Mangaia	Mangaia	76.1	79.1	96.9	94.0	*	No MDA	92.7	MDA stopped
Mauke	Mauke	73.7	83.5	97.0	90.7	*	No MDA	97.3	MDA stopped
Mitiaro	Mitiaro	57.0	86.2	95.7	95.7	*	No MDA	94.3	MDA stopped
Palmerston	Palmerston	100.0	*	95.8	93.8	*	No MDA	86.3	MDA stopped
Rarotonga	Rarotonga	84.3	75.3	96.0	98.2	*	No MDA	94.4	MDA stopped
Manihiki	Manihiki	83.5	86.8	94.5	95.0	*	No MDA	97.8	MDA stopped
Nassau	Nassau	*	85.7	88.4	92.8	*	No MDA	*	MDA stopped
Penrhyn	Penrhyn	100.0	100.0	100.0	100.0	*	No MDA	100.0	MDA stopped
Pukapuka	Pukapuka	77.8	81.4	92.2	92.8	*	No MDA	93.8	MDA stopped
Rakahanga	Rakahanga	63.5	49.3	94.0	95.2	*	No MDA	96.7	MDA stopped

*Data not available

### B-Survey (2002)

In the B-Survey, a total of 2025 people were tested for Ag. Of the 11 islands surveyed, 8 islands showed no ICT positivity, and in other 3 islands, the Ag prevalence ranged from 0.13 to 4.00%. The overall Ag prevalence for the all surveyed islands was 0.59%, compared to 9.66% in the A-Survey. The results clearly suggested that the MDA program had been as effective as expected, although two islands still showed > 1.0% Ag prevalence (Table 2).

### C-Survey (2005)

The detailed results of the C-Survey are presented in Table 4. The number of people sampled in the three EUs differed—including 1000 individuals in Rarotonga and 900 in the southern group (both adequate for a statistical definition of < 1% prevalence of ICT-positives) but only 502 individuals in the northern group (not adequate for statistical determination of a < 1% prevalence of ICT-positives). The findings from this C-Survey showed that the Ag prevalence in the EU of *Rarotonga* was only 0.20%. Hence, the IU could proceed to a D-Survey of children to assess the potential interruption of LF transmission. The Ag prevalence in the southern group of islands was 2.56% (not yet meeting the < 1% criterion); the northern group was not sufficiently sampled, and even in those who were sampled, the Ag prevalence was 1.79%, indicating that this EU too had *not* achieved the criteria required for proceeding to the D-Survey. Hence, it was decided to implement one more MDA (sixth round of MDA) in 2006 both in the northern and the southern groups of islands. The EU Rarotonga was also included for MDA, although the Ag prevalence was less than the critical level of 1.0%. In addition, all individuals who had been identified as Ag-positive (Table 4) were treated in 2005.

**Table 4:** Details of C-Survey, 2005

EU Rarotonga			EU southern group			EU northern group		
Village surveyed	No. of people surveyed	% Ag positive	Island surveyed	No. of people surveyed	% Ag positive	Island surveyed	No. of people surveyed	% Ag positive
Tupapa/Maraerenga	200	0.00	Atiu	150	0.00	Pukapuka	200	3.50
Takuvaine/Tutakimoa	200	0.50	Aitutaki	300	7.33	Penrhyn	100	0.00
Ruatonga/A Vaitu	200	0.50	Mauke	150	0.00	Manihiki	100	0.00
Pokoinu/Nikao	200	0.00	Mangaia	200	0.50	Rakahanga	100	2.00
Takitumu	200	0.00	Mitiaro	100	0.00	Suwarrow	2	0.00
Puaikura	200	0.00	–	–	–	–	–	–
Total	1000	0.20	–	900	2.56	–	502	1.79

### D-Survey (2007)

In the D-Survey, of the 4415 people who were surveyed, 12 were found to be Ag-positive, and the national Ag prevalence was 0.27%. Ten of the 12 islands showed 0% Ag prevalence, and only 2islands—Pukapuka and Mitiaro—accounted for all 12 positive individuals. Based on these results, the national MDA program was stopped, and all 12 Ag-positive individuals were treated with single-dose DEC + ALB.

### Test and treat strategy in Pukapuka and Mitiaro (2006–2012)

All the people found positive for Ag were treated with DEC + ALB. For example, five people were detected with Ag during 2009–2010, and all of them were treated. The follow-up survey conducted both in the islands in 2012 tested as many individuals as possible (more than 400 in each island). None was Ag-positive.

### Post-MDA surveillance surveys (2013–2014)

In the post-MDA surveillance survey conducted in 2013–2014, there were 2903 individuals in ten islands tested, including 300 children in Rarotonga and 275 children in Aitutaki (Table 5). Only one person (an adult) was found positive for Ag, and he was from Mangaia island. No Ag-positive individual was found in the other 11 islands. The national Ag prevalence rate could be calculated at 0.03%, against the critical cutoff value of 1.0% prevalence in *Aedes*-transmitted LF [7].

**Table 5:** Post-MDA surveillance survey (2013–2014)

Island (implementation unit)	Evaluation unit	Number tested^#^	Number positive
Aitutaki	Aitutaki	275	0
Atiu	Atiu	*	0
Mangaia	Mangaia	*	1
Mauke	Mauke	*	0
Mitiaro	Mitiaro	122	0
Palmerston	Palmerston	*	0
Rarotonga	Rarotonga	300	0
Manihiki	Manihiki	*	0
Nassau	Nassau	66	0
Penrhyn	Penrhyn	*	0
Pukapuka	Pukapuka	388	0
Rakahanga	Rakahanga	*	0

*Data no longer available (entire island population tested)

^#^In Aitutaki and Rarotonga, children were tested

### MMDP

As per hospital records and observations by health workers, there was only one individual affected with chronic filarial disease (lymphoedema) and no filarial hydrocele patients in the country. The patient with lymphoedema has been given training on clinical management of her disease.

## Discussion

The history of LF in the Cook Islands records a high prevalence of disease, which is known to inflict considerable social and economic burden on the affected communities [8, 9]. In some islands, nearly half of the population was microfilaremic, and while over the years, the prevalence of infection had decreased, still there were pockets of infection in several parts of the country at the time when PacELF began in 1999. Aitutaki and Mauke, for example, recorded an Mf rate of 3.2 and 4.3%, respectively, in 1992 (Table 1), and such pockets of infection tend to persist for prolonged times [10, 11], unless concerted efforts are made to eliminate the infection.

Along with other countries, the Cook Islands joined the PacELF in 1999, adopting its strategies and preventive chemotherapy opportunities to eliminate LF [2]. Implementation of the LF elimination program in countries with populations spread across many islands, as in the Cook Islands, is logistically difficult. However, the MOH was able to overcome these logistic difficulties and, as seen in the data presented above, eliminate LF as a public health problem following the standard MDA strategy, except in Pukapuka and Mitiaro where foci of persistent infection required intensive treatment and surveillance using a “test and treat” approach to eliminate the infection. The success of the Cook Islands serves as an example for other island countries in the region to effectively plan and implement MDA and evaluation activities and to achieve high treatment coverage even in remote and small islands.

The other notable feature of the program is its sound epidemiologic and pro-active approach. For example, it identified and sampled additional age groups and adults whenever adequate numbers of children were not available for the assessment of the prevalence of antigenemia. Also, it conducted additional surveys and implemented test and treat strategies to remove the persistent infection in some islands to ensure no resurgence of infection.

The results of the final Ag survey, conducted in 2013–2014, showed that the Ag prevalence was 0% in 11 out of 12 islands, and the “national Ag prevalence” was only 0.03%. Such an achievement is particularly noteworthy, as the local vector is an *Aedes* mosquito, which has the capacity to prolong the endgame phase of LF elimination programs [10, 11] due to their higher vectorial capacity [3, 12]. Elimination of LF in the Cook Islands will stand as a very positive example to other countries in the region and elsewhere, where *Aedes* species act as vectors. With this likely total interruption of transmission, combined with the current good socio-economic conditions in the Cook Islands, the resurgence of LF is extremely unlikely. However, the program recognizes the need to be alert to post-elimination surveillance strategies now being established by the WHO and based on multi-disease serologic monitoring initiatives.

The Cook Islands is now almost completely free from any chronic LF disease burden, principally because of the gradual decline of LF over the prior few decades but also because of the current MDA program and the MMDP efforts required to meet the WHO criteria for the elimination of the disease as a public health problem [13].

## Conclusion

The epidemiologic and treatment data presented in this paper and the points discussed above formed the basis of the LF elimination dossier prepared by the Cook Islands for submission to the WHO Western Pacific Regional Office in 2016 as evidence for the successful LF elimination program. That same year, after due diligence to the dossier, the persistence and energies of the MOH were rewarded with WHO’s official acknowledgement of the successful validation of elimination of LF as a public health problem from the Cook Islands (http://www.wpro.who.int/mediacentre/releases/2016/20161010a/en/)!

## Abbreviations

Ag: Antigen
ALB: Albendazole
DEC: Diethylcarbamazine
EU: Evaluation unit
ICT: Immunochromatographic card test
IU: Implementation unit
LF: Lymphatic filariasis
MDA: Mass drug administration
Mf: Microfilaria
MOH: Ministry of Health
PacELF: Pacific Programme to Eliminate Lymphatic Filariasis
WHO: World Health Organization

## References

[1] Human Filariasis SM (1976). A global survey of epidemiology and control.

[2] WHO (2006). The PacELF way. Towards the elimination of lymphatic filariasis from the Pacific. 1999–2005.

[3] Pichon G (2012). Limitation and facilitation in the vectors and other aspects of the dynamics of filarial transmission: the need for vector control against Anopheles-transmitted filariasis. Ann Trop Med Parasitol.

[4] Ottesen EA (2000). The global programme to eliminate lymphatic filariasis. Trop Med Int Health.

[5] Steel C, Ottesen EA, Weller PF, Nutman TB (2001). Worm burden and host responsiveness in Wuchereria bancrofti infection: use of antigen detection to refine earlier assessments from the South Pacific. Am J Trop Med Hyg.

[6] Steel C, Guinea A, Ottesen EA (1996). Evidence for protective immunity to bancroftian filariasis in the Cook Islands. J Infect Dis.

[7] WHO (2011). Global programme to eliminate lymphatic filariasis. Monitoring and epidemiological assessment of mass drug administration. A manual for national elimination programmes.

[8] Person B, Addiss D, Bartholomew LK, Meijer C, Pou V, Gonzalvez G, Borne BV (2008). “Can it be god does not remember me”: a qualitative study on the psychological distress, suffering, and coping of Dominican women with chronic filarial lymphedema and elephantiasis of the leg. Health Care Women Int.

[9] Ramaiah KD, Das PK, Michael E, Guyatt H (2000). The economic burden of lymphatic filariasis in India. Parasitol Today.

[10] Lau CL, Won KY, Becker L, Soares Magalhaes RJ, Fuimaono S, Melrose W, Lammie PJ, Graves PM (2014). Seroprevalence and spatial epidemiology of lymphatic filariasis in American Samoa after successful mass drug administration. PLoS Negl Trop Dis.

[11] Lau CL, Won KY, Lammie PJ, Graves PM (2016). Lymphatic filariasis elimination in American Samoa: evaluation of molecular xenomonitoring as a surveillance tool in the endgame. PLoS Negl Trop Dis.

[12] Southgate BA (1992). The siginificance of low density microfilaraemia in the transmission of lymphatic filarial parasites. J Trop Med Hyg.

[13] World Health Organization. Validation of elimination of lymphatic filariasis as a public health problem. Geneva; 2017. http://apps.who.int/iris/bitstream/10665/254377/1/9789241511957.

